# Using fine‐scale spatial genetics of Norway rats to improve control efforts and reduce leptospirosis risk in urban slum environments

**DOI:** 10.1111/eva.12449

**Published:** 2017-02-23

**Authors:** Jonathan L. Richardson, Mary K. Burak, Christian Hernandez, James M. Shirvell, Carol Mariani, Ticiana S. A. Carvalho‐Pereira, Arsinoê C. Pertile, Jesus A. Panti‐May, Gabriel G. Pedra, Soledad Serrano, Josh Taylor, Mayara Carvalho, Gorete Rodrigues, Federico Costa, James E. Childs, Albert I. Ko, Adalgisa Caccone

**Affiliations:** ^1^ Department of BiologyProvidence CollegeProvidenceRIUSA; ^2^ Department of Ecology & Evolutionary BiologyYale UniversityNew HavenCTUSA; ^3^ Centro de Pesquisas Gonçalo MonizFundação Oswaldo CruzMinistério da SaúdeSalvadorBrazil; ^4^ Centro de Controle de ZoonosesSecretaria Municipal de SaúdeMinistério da SaúdeSalvadorBrazil; ^5^ Instituto de Saúde ColetivaUniversidade Federal da Bahia, UFBASalvadorBrazil; ^6^ Department of Epidemiology of Microbial DiseaseYale School of Public HealthNew HavenCTUSA

**Keywords:** epidemiology, favela, individual‐based sampling, intervention, landscape genetics, population genetics, public health, reservoir host, spatial scale, urban ecology, vector control

## Abstract

The Norway rat (*Rattus norvegicus*) is a key pest species globally and responsible for seasonal outbreaks of the zoonotic bacterial disease leptospirosis in the tropics. The city of Salvador, Brazil, has seen recent and dramatic increases in human population residing in slums, where conditions foster high rat density and increasing leptospirosis infection rates. Intervention campaigns have been used to drastically reduce rat numbers. In planning these interventions, it is important to define the eradication units ‐ the spatial scale at which rats constitute continuous populations and from where rats are likely recolonizing, post‐intervention. To provide this information, we applied spatial genetic analyses to 706 rats collected across Salvador and genotyped at 16 microsatellite loci. We performed spatially explicit analyses and estimated migration levels to identify distinct genetic units and landscape features associated with genetic divergence at different spatial scales, ranging from valleys within a slum community to city‐wide analyses. Clear genetic breaks exist between rats not only across Salvador but also between valleys of slums separated by <100 m—well within the dispersal capacity of rats. The genetic data indicate that valleys may be considered separate units and identified high‐traffic roads as strong impediments to rat movement. Migration data suggest that most (71–90%) movement is contained within valleys, with no clear source population contributing to migrant rats. We use these data to recommend eradication units and discuss the importance of carrying out individual‐based analyses at different spatial scales in urban landscapes.

## 1 Introduction

Over the last century, there has been a marked increase in the proportion of the human population living in urban areas, from <14% in 1900 to more than 54% in 2015 (UN; WHO). As a result, during this period urban landscapes have been expanding while human population densities have increased. Despite this trend, urban habitat has generally been considered depauperate in biodiversity and received little attention from ecologists and population geneticists relative to its increasing scale on the landscape. However, urban landscapes can have large impacts on wildlife (Mcdonald, Kareiva, & Forman, 2008). The negative impacts are well‐appreciated, and declines in population size and barriers to movement have been documented in species sensitive to habitat modification (Geslin et al., 2016; Krausman, Derbridge, & Merkle, 2008; Noël, Ouellet, Galois, & Lapointe, 2007; Wood & Pullin, 2002). However, there are a number of species that persist and even thrive in urban habitats. Some species can inhabit small patches of remnant habitat within an urbanized area, while other species characterized as “urban exploiters” can take advantage of the modified habitat and increased food resources (Blair, 2001; Hulme‐Beaman et al., 2016; McKinney, 2002).

To date, very few studies have investigated how species that thrive in urban habitats disperse through these heavily developed landscapes. Importantly, many of the species best adapted to living alongside urban human populations are considered nuisance species capable of damaging property, ruining food stocks, and transmitting disease (Lyytimäki, Petersen, Normander, & Bezák, 2008). In USA, the costs of rats to agriculture alone are estimated at 19 billion dollars (Pimentel, Zuniga, & Morrison, 2005). The global costs of eradication and control efforts are uncertain but considerable, particularly in urban habitats. It is critical to understand how the urban landscape shapes dispersal patterns in urban species—particularly rat pests—so that public health and urban infrastructure officials can pursue strategies that control or restrict the movement of these species.

The Norway rat (*Rattus norvegicus*, also called the brown rat) is an urban exploiting pest species that has expanded to a near‐global range over the last 300 years. Originally native to forests and brushy habitat in northern China, *R. norvegicus* is now most commonly associated with human‐dominated landscapes and thrives in areas of high human population density (Feng & Himsworth, 2013). As a result, *R. norvegicus* is one of the most important nuisance species globally, responsible for significant agricultural losses, negative impacts on native ecosystems, and disease transmission as the reservoir host of major zoonotic pathogens (Antoniou et al., 2010; Capizzi, Bertolino, & Mortelliti, 2014; Pimentel et al., 2005). In particular, urban areas with low socioeconomic conditions and high human densities in developing countries face the greatest risk of rat‐associated diseases that are linked with increasing rat populations (Gratz, 1994; Himsworth, Parsons, Jardine, & Patrick, 2013; Ko, Reis, Dourado, Johnson, & Riley, 1999; Lau, Smythe, Craig, & Weinstein, 2010). The rapid increase in people living in slum communities with subadequate housing, poor sanitation, and limited access to health care is considered to be one of the major challenges to public health (Riley, Ko, Unger, & Reis, 2007; Sclar, Garau, & Carolini, 2005; Un‐Habitat, 2004).

The city of Salvador, Brazil (pop. 2.9 million), has seen a 500% increase in human population size over the last 60 years, with most new settlement occurring in urban slum, or favela, neighborhoods within the city (Reis et al., 2008). These densely populated and low socioeconomic areas often have limited access to municipal services and experience derelict housing and poor sanitation (Felzemburgh et al., 2014; Hagan et al., 2016; Reis et al., 2008). These conditions support rodent activity and *R. norvegicus* infestation with several pathogens that cause spillover infections to humans (Costa et al., 2014, 2015; Glass, Childs, Korch, & LeDuc, 1989; Ko, Goarant, & Picardeau, 2009). There has also been a corresponding rise in the incidence of leptospirosis in this area—a potentially life‐threatening disease affecting respiratory, renal, and liver functioning in infected humans (Ko et al., 1999; Lau et al., 2010). The disease is zoonotic and caused by spirochete bacteria in the same family as the agents of syphilis and Lyme disease. Rats are chronically infected with the bacteria, and the infectious leptospires are shed in rat urine creating an environmental reservoir for the pathogen (Costa et al., 2015; Lau et al., 2010). The leptospires can survive in soil and aquatic environments for weeks to months, leading to increased human outbreaks during the rainy season.

In cities throughout Brazil, which, as in Salvador, have annual rainfall‐associated outbreaks of leptospirosis, public health officials have initiated intervention campaigns to sharply reduce rat numbers within these urban habitats at increased risk. Control efforts consist of rodenticide poisoning and environmental interventions (e.g., covering free‐flowing sewers) in areas with high leptospirosis incidence. However, rat numbers rebound quickly with population sizes reaching pre‐intervention densities within six months (de Masi, Vilaça, & Razzolini, 2009). Therefore, data on rat dispersal patterns are critical for designing more efficient rodent control programs, including where to target resources for intervention campaigns across large sections of urban habitat to impede re‐colonization of treated areas (Abdelkrim, Pascal, & Samadi, 2007). Groups of vector organisms that are in close proximity and interconnected are defined as “eradication units.” Pest populations within these units must be controlled simultaneously to prevent reinvasion and augment long‐term success of control campaigns (Abdelkrim et al., 2007; Robertson & Gemmell, 2004; Russell et al., 2010).

Distinguishing dispersal events from population rebound due to animals surviving interventions is critical for effective control. While data on movement patterns are difficult to gather efficiently, or ethically untenable in the case of a zoonotic disease vector, using traditional mark–recapture and radio tracking methods (Davis, Emlen, & Stokes, 1948; Fenn, Tew, & Macdonald, 1987; Glass et al., 1989; Peakall, Ebert, Cunningham, & Lindenmayer, 2006; Taylor, 1978; Taylor & Quy, 1978; Wilson, Efford, Brown, Williamson, & McElrea, 2007), genetic data can be used to estimate rates of gene flow and provide valuable insights into the degree of connectivity, routes of dispersal, and spatial networks linking organisms across a complex urban landscape (Munshi‐South, 2012). Researchers have used genetic data to determine relatedness of rats within prescribed locations and to estimate connectivity across urban areas (Gardner‐Santana et al., 2009; Kajdacsi et al., 2013). However, even these genetic studies suffer from modest sample sizes (*n* = 146–277 rats) distributed across large urban regions, where low sampling density limits the resolution of spatial genetic data and restricts our ability to detect patterns across complex urban landscapes. Moreover, the distribution of sampled rats tends to be clumped at a limited number of sites, reducing the spatial coverage of the study to the areas sampled. While genetic data are a valuable proxy for individual movement and dispersal, it should be noted that these data are providing insight into the subset of all dispersal events that result in breeding (Lowe & Allendorf, 2010). This functional connectivity may not represent all individual movements, for which local factors may inhibit or promote assimilation into the local breeding cohort (Fraser, Banks, & Waters, 2015).

In this study, we evaluated genetic connectivity among more than 700 urban Norway rats captured from Salvador, Brazil, to identify eradication units that will be used to direct future rat control campaigns in Salvador. We also used our large sampling numbers to advance the spatial scope and analytical framework beyond what has been used to date in urban population genetics. We focused on three hierarchical spatial scales of analyses, from a local slum neighborhood to city‐wide sampling, to evaluate how spatial genetic patterns differ based on the scale and resolution of sampling. We combined geographically distributed rat genotype data with individual‐based analyses, providing much greater resolution of spatial genetic patterns than could be afforded from combining individuals in arbitrarily assigned sampling sites—particularly at the fine spatial scales that correspond to heterogeneous urban habitat structure. We use spatially explicit analyses that increase our power to detect genetic structure and link this directly to spatial location and connections on the landscape (Guillot, Santos, & Estoup, 2008; Jombart, Devillard, Dufour, & Pontier, 2008). We also use two methods that are free from the limiting assumptions of many population genetic models and that bypass the issue of nonindependence among pairwise genetic distances (Galpern, Peres‐Neto, Polfus, & Manseau, 2014; Jombart, Devillard, & Balloux, 2010).

**Figure 1:**
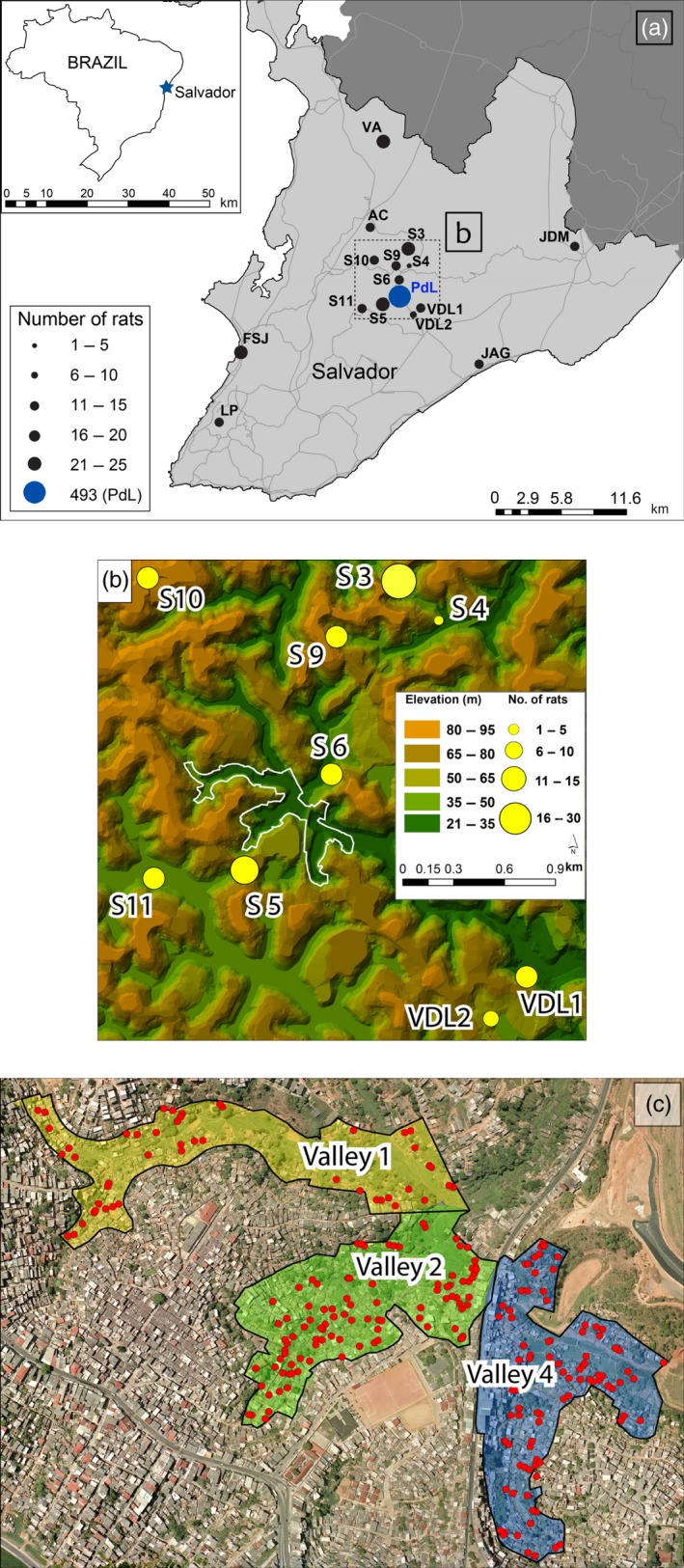
(a) Map of the study area within Salvador, Brazil. Black circles (and size) indicate sampled sites and the number of rat genotypes analyzed at each site at the city‐wide scale. (b) The intermediate‐scale analyses included nine satellite sites within 1 km of the Pau da Lima slum. Yellow dots are sampled sites and are scaled to the number of rats. (c) An aerial image of the Pau da Lima slum, with the three main valleys delineated. Red dots represent the sampling locations of the 493 rats analyzed at this slum scale. Note that in some cases, multiple rats were collected from the same geographic coordinate, corresponding to a building or structure

To achieve these goals, we collected rats from across the city of Salvador, with an emphasis on Pau da Lima, a slum neighborhood that has been the focus of long‐term epidemiological studies related to leptospirosis (Figure 1) (Felzemburgh et al., 2014; Hagan et al., 2016; Reis et al., 2008). We genotyped rats at 16 variable microsatellite loci, and used these data to identify spatial genetic patterns and levels of connectivity that promote or impede the movement of rats at three spatial scales, ranging from city wide to areas within and around Pau da Lima. We also used spatial genetic structure to delineate practical eradication units for future interventions. Our study takes advantage of the largest sample size ever included in a study of urban rat ecology and their spatial genetic structure. This includes many samples collected within the dispersal range observed for *R. norvegicus*—distances within which any potential genetic subdivision must be caused by ecological or habitat features rather than movement abilities.

## 2 Materials and Methods

### 2.1 Study area and sampling

We sampled 706 rats at 19 geographically separate regions across the city of Salvador using both live and snap traps (Figure 1a). These 706 rats greatly expand the number of areas and samples analyzed by Kajdacsi et al. (2013). Trap sites were geo‐referenced, and tail tissue was collected and stored in ethanol at −80°C until DNA was extracted. Nearly 60% of the human population of Salvador lives within slum developments (Moreira and Pereira 2008). For that reason, we sampled most intensively across one of them, Pau da Lima. This slum is located in the center of Salvador and includes four connected valleys (valleys 1–4; Figure 1c). Pau da Lima was selected as the smallest scale with increased sampling based on the high density of rats and the high incidence human leptospirosis (Costa et al., 2014; Felzemburgh et al., 2014). The samples in this study were collected from three of the valleys (valleys 1, 2, and 4; Figure 1); Valley 3 has not been part of the larger long‐term eco‐epidemiological study. The 493 samples collected from within Pau da Lima constituted the finest spatial scale, referred to as “slum scale.” The next spatial scale included 122 samples collected from eight “satellite” sites located within 1 km of Pau de Lima; we refer to the combination of these “satellite” locations and Pau da Lima ones as “intermediate scale.” Lastly, 91 rats were collected beyond this intermediate scale in seven additional areas of the city (2–21 km from Pau da Lima); we refer to this scale as “city wide” in our three‐tier hierarchy of sampling (Figure 1).

### 2.2 DNA extraction and genotyping

DNA was extracted from 2–5 mm of tail tissue using standard kit‐based extraction protocols (Qiagen and ZyGEM). We then amplified 16 microsatellite loci previously identified as polymorphic in *Rattus* spp. using a touchdown PCR protocol and reaction conditions following Kajdacsi et al. (2013). PCR amplicons were identified using capillary electrophoresis on an ABI 3730 DNA sequencer. GeneMarker software was used to score alleles, and Microsatellite Toolkit v3.1 (Park 2001) was used to check for scoring errors.

Although most of our analyses have no requirements of Hardy–Weinberg equilibrium and the loci were selected based on chromosome location to ensure linkage equilibrium among loci, we evaluated any deviations from these two common population genetic models. Independence of loci was evaluated by testing for linkage disequilibrium in FSTAT v2.9.3 (Goudet, 1995). Hardy–Weinberg equilibrium (HWE) for each locus was also estimated, with significance estimated using 10,000 randomizations. Significance values were adjusted for multiple testing using the sequential Bonferroni method (Holm 1979). Null allele frequencies were obtained using Microchecker v2.2.3 (van Oosterhout, Hutchinson, Wills, & Shipley, 2004). Loci exhibiting null allele frequencies >0.20 were excluded from later analyses (Chapuis & Estoup, 2007). Results for these marker tests and summary statistics can be found in the online Supporting Information.

### 2.3 Individual‐based genetic analyses

Rather than arbitrarily grouping rats for population‐level genetic analyses, we took advantage of our high‐resolution sampling (i.e., many rats over a large area) to perform individual‐based analyses. Using individuals as the unit of analysis rather than populations allows for the evaluation of much finer‐scale genetic patterns because information is not lost by averaging within groups (Richardson, Brady, Wang, & Spear, 2016). Most importantly, individual‐based approaches can be more powerful in detecting small‐scale genetic patterns (Luximon, Petit, & Broquet, 2014; Prunier et al., 2013) and are at least complementary to the population‐based models that most population genetic analyses are based on. We used the program SPAGeDi to calculate genetic distances between every pair of individuals in the dataset (Hardy & Vekemans 2002). To look for a correlation between genetic differentiation and geographic distance between individuals, we performed a Mantel test of matrix correlation with 10,000 permutations using the vegan package within R (Oksanen et al., 2016).

### 2.4 Spatially explicit ordination of the genetic data (spatial PCA)

Principal component analyses (PCA) are an effective method to create new synthetic variables that maximize the variance among genotypic data (Patterson, Price, & Reich, 2006). More recently, extensions of PCA have been developed to include spatial data in this ordination approach (Jombart et al., 2008). We performed spatial principal component analysis (sPCA) within the adegenet package of R (Jombart et al., 2016). We selected the parameters used based on initial insights from eigenvalue decomposition and recommendations from the program authors. We used an inverse distance‐weighted connection network with no exponent because of the large number of samples and a minimum distance of one to maximize sample inclusion.

sPCA uses the spatial coordinates of each sample to estimate the degree and direction of spatial autocorrelation in the genetic data using Moran's *I* index. Global genetic structure evaluates the level of positive spatial autocorrelation or whether a cline exists where genetic similarity between individuals decreases as the distance between samples increases. Strong local‐scale structure indicates negative spatial autocorrelation, and sharp genetic breaks between samples located close together. We used the eigenvalue variance and spatial components of the sPCA analysis to select the number of global and local axes to retain and visualize (Jombart et al., 2016). We used the sPCA‐specific multivariate significance test to determine the strength of the global and local genetic structure across genotypes. These tests use Moran eigenvector maps (MEM) created by decomposing the connection network matrix and estimating the r^2^ correlation coefficients of alleles from the global and local MEMs. The significance was determined using simulated *p*‐values across 10,000 permutations where each row in the allele frequency matrix is randomized (Jombart et al., 2008). This allows us to test the null hypothesis that allele frequencies are distributed randomly across the connection network, with no spatial structure.

### 2.5 Discriminant analysis of genetic structure

We used discriminant analysis of principal components (DAPC) to evaluate (A) the degree of genetic similarity among individuals in the dataset and (B) the patterns of genetic similarity among individuals from different areas of Salvador and within Pau da Lima. DAPC uses coefficients of allele loadings in linear combinations to maximize the between‐groups variance while minimizing within‐group variances in these loadings (Jombart et al., 2010). Studies using simulated and empirical data have found that DAPC performs as well or better than other individual‐based clustering methods (e.g., structure; Pritchard, Stephens, & Donnelly, 2000), particularly when complex processes are operating (Jombart et al., 2010; Klaassen, Gibbons, Fedorova, Meis, & Rokas, 2012). Prior to DAPC analyses, we added location tags for each rat corresponding to the geographic area that it was collected to help visualize the patterns in discriminant function space. We used guidelines in the *adegenet* package in R 2.14 to conduct DAPC and k‐means clustering while retaining the best supported numbers of principal components and eigenvalues (R Development Core Team 2015; Jombart et al., 2016).

### 2.6 Mapping spatially explicit genetic discontinuity

We used a fourth method that is based on Hardy–Weinberg equilibrium to look for convergence among different approaches characterizing spatial genetic structure. This approach, implemented in the Geneland package in R, is a Bayesian model using both genotypic data and spatial coordinates to identify areas of genetic continuity, where Hardy–Weinberg disequilibrium is minimized (Guillot, Mortier, & Estoup, 2005). Results are then visualized using Voronoi tessellation and membership coefficients. Studies indicate that Geneland performs better than other Bayesian clustering approaches in terms of correctly assigning individuals into groups (François & Durand, 2010). We used 100,000 Markov chain Monte Carlo iterations of the model with a thinning interval of 100. Uncorrelated allele frequencies and geographic coordinates without error were used to parameterize the model run. We used posterior probabilities to determine the number of genetic groups that were best supported across these runs. Three independent runs were used to confirm convergent results after the 100,000 iterations.

### 2.7 Moran's eigenvector mapping to account for spatial nonindependence

Much criticism has been raised regarding population genetic analyses based on pairwise distance data (e.g., genetic and geographic distance), which are often used in Mantel tests of isolation by distance (Legendre, Fortin, & Borcard, 2015). This is because the pairwise values are not independent when each site is associated with multiple distances in the matrix. Moran's eigenvector mapping (MEM) has been developed to evaluate the spatial relationship among locations for any variable of interest. The MEM approach creates new uncorrelated (i.e., orthogonal) variables describing the spatial autocorrelation, and hence spatial relationships, among sampling sites (Dray, Pierre, & Peres‐Neto, 2006).

We performed a MEM analysis to identify spatial genetic patterns among rats using our geographic and genotype data within the MEMGENE package of R (Galpern et al., 2014). This approach produces new spatially independent variables that summarize the spatial relationship of genetic differences between sampled rats (Peres‐Neto & Galpern, 2015). The proportion of shared alleles was used as the measure of genetic distance between individual rats (Bowcock et al., 1994). We extracted new MEM variables and visualized patterns via spatially explicit mapping of these MEM variable scores.

### 2.8 Migration rates across Salvador

We estimated the rates of recent and ongoing genetic migration between the different sampling areas using the default parameters within the program BayesAss v3.0 (Wilson & Rannala, 2003). This approach uses Bayesian Markov chain Monte Carlo resampling to estimate posterior probabilities of ancestry within the last several generations. It also allows deviations from Hardy–Weinberg equilibrium, while assuming that loci are in linkage equilibrium and null alleles are not present. Immigrants are assumed to exhibit short‐term linkage disequilibrium relative to the recipient population. This analysis estimates migration for all pairwise connections (i.e., [*n*(*n*‐1)/2], and to make this computationally feasible, we grouped the 706 rats into 28 geographic areas based on topography and distance from other sampling areas (Fig. S1).

## 3 Results

### 3.1 Spatially explicit ordination of the genetic data (spatial PCA)

**Figure 2:**
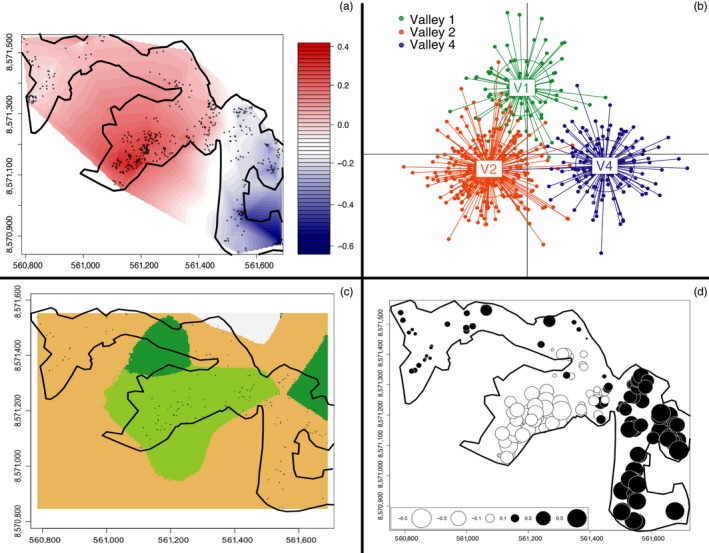
Analyses from the smallest spatial scale within the Pau da Lima slum show strong and consistent genetic segregation between valleys 2 and 4. Points in panels a, c, and d are the 493 spatially referenced rat sampling sites, overlaid with the outline of Pau da Lima (x‐ and y‐axes are spatial coordinates). (a) Spatially explicit principal component (sPCA) vector scores interpolated across the sampling area show a sharp genetic break between valleys 2 and 4, while Valley 1 is admixed but more similar to Valley 2. (b) Discriminant analysis of principal components (DAPC) within Pau da Lima shows genetic separation between all three valleys in discriminant function space; each rat genotype is represented by a point with a line connecting it to the centroid ellipse of that group. Axes are discriminant function variables. (c) The spatial Bayesian model also indicates strong divergence between valleys 2 and 4, represented by the different tessellation colors. Each color unit has at least one rat contained within. (d) Consistent with the other analyses, Moran's eigenvector mapping (MEM) shows clear divergence between valleys 2 and 4, with circle color and size representing genetic similarity along the first MEM variable axis. Valley 1 is more similar to Valley 4 in the Bayesian and MEM analyses

**Figure 3:**
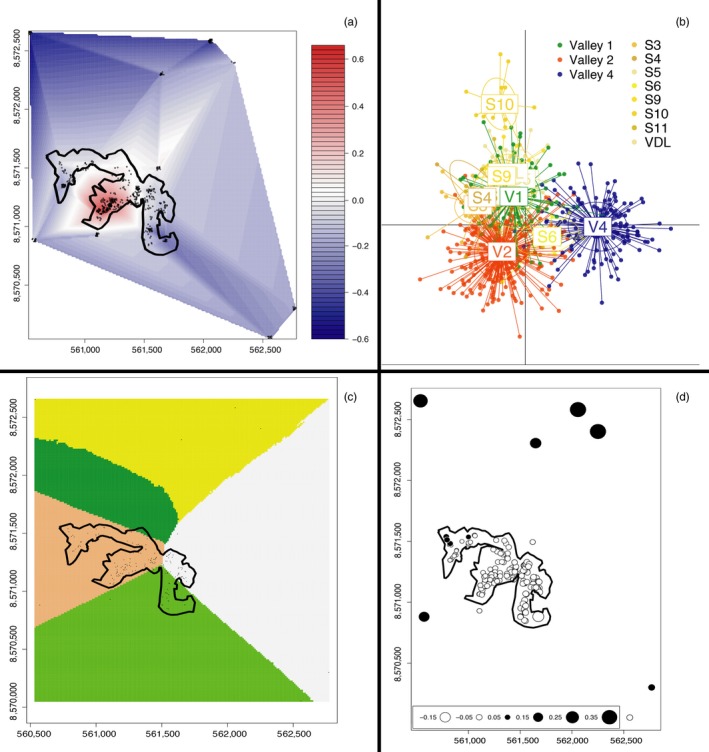
Analyses from the intermediate spatial scale, including Pau da Lima (black polygon outline) and all satellite sites within 1 km, show strong genetic divergence between valleys 2 and 4, with the exception of the MEM (D). Points in a, c, and d are the 614 spatially referenced rat samples. (a) sPCA vector scores interpolated across the sampling area show a sharp genetic break between Valley 2 and the rest of the sampling area, while Valley 1 is admixed but more similar to Valley 4. (b) DAPC indicates that most of the sampling sites cluster together, with the exception of clear divergence of Valley 4 and satellite site S10 in discriminant function space. (c) The spatial Bayesian model also shows strong divergence between valleys 2 and 4, represented by the different tessellation colors. At this scale, valleys 1 and 2 are grouped together and genetically distinct from all other sample areas. (d) MEM at the intermediate scale is the only analysis that does not exhibit divergence between valleys 2 and 4, as indicative by symbol color and size. Most satellite sites away from Pau da Lima appear as separate genetic group (black circles)

**Figure 4:**
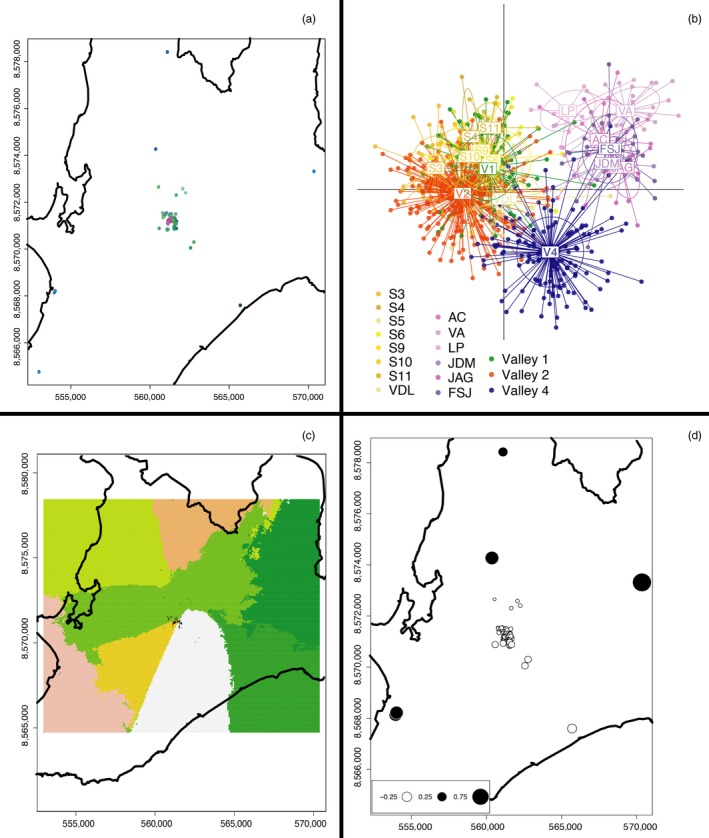
Analyses at the largest city‐wide scale from all sites in Salvador also show strong and consistent genetic segregation between valleys 2 and 4. Most outlying sampling sites around the city cluster together relative to the Pau da Lima area. Points in a, c, and d are the 706 spatially referenced rat sampling sites. (a) The sPCA color plot displays genetic similarity as similarly colored points; the interpolated scores used in Figures 2 and 3 are less tractable at this large scale. Salvador‐wide data exhibit a sharp genetic break between valleys 2 and 4, while the outlying sites are clustered as one group. (b) DAPC indicates three primary genetic groups—Valley 4, all outlying sites, and the rest of the Pau da Lima and nearby satellite sites. (c) The spatial Bayesian model shows strong divergence between all three valleys within Pau da Lima, while the outlying sites are mostly represented by a unique genetic group based on geography (represented by the different tessellation colors). (d) MEM exhibits strong divergence between the outlying sites around Salvador and the Pau da Lima area and their nearby satellite sampling areas

We found significant global structure in the rat genetic data at all three spatial scales (slum scale *p *=* *.002, intermediate scale *p *=* *.008, city‐wide scale *p *=* *.002). At each scale, Valley 2 within Pau da Lima (Figure 1b) was clearly identified as genetically dissimilar from the adjacent valleys (Figures 2a, 3a, and 4a). At the slum scale within the Pau da Lima community, the PCA‐based genetic signature of Valley 1 is intermediate between valleys 2 and 4, a pattern also seen in the DAPC analysis (Figure 2a,b). There were no significant patterns of local‐scale genetic structure at any of the three spatial scales, meaning that there was no negative autocorrelation in the genetic data across the study area (city‐wide scale *p *=* *.23, intermediate scale *p *=* *.19, slum scale *p *=* *.27). One to three principal components explained the most variation at all scales, and those eigenvalues were used to plot the sPCA data (Jombart et al., 2016). These patterns of global structure can result from a number of processes, including isolation of patches, adaptive divergence in response to spatially autocorrelated natural selection, or clinal isolation by distance (Jombart et al., 2008; Wagner & Fortin, 2005). We detected no significant local genetic structure with sPCA, which can arise when individuals avoid breeding with other members of their population/deme (e.g., inbreeding avoidance, repulsion), or where adaptive divergence occurs at small spatial scales in response to sharp gradients in natural selection (Richardson, Urban, Bolnick, & Skelly, 2014).

### 3.2 Discriminant analysis of genetic structure

For each DAPC run, we retained the number of principal components necessary to explain between 80 and 90% of the variance, which ranged from 75 to 100 PCs. There was clear genetic grouping of individuals across all three spatial scales, including the finest scale within Pau da Lima. At the slum scale, all three valleys within Pau da Lima were genetically distinct, with more overlap between valleys 1 and 2 than Valley 4 (Fig. 2b). At the intermediate scale, Valley 4 was still clearly distinct, while valleys 1 and 2 overlapped extensively with the nearby satellite populations, with the exception of site S10 (Figure 3b). When all 706 rats across Salvador were considered at the city‐wide scale, Valley 4 again was distinct from the rest of Pau da Lima and the nearby satellite sampling areas, while the outlying sampled areas across Salvador were genetically segregated from the three Pau da Lima valleys (Figure 4b).

### 3.3 Mapping spatially explicit genetic discontinuity

Results from each Geneland run converged between 10,000 and 40,000 iterations, well before the completed 100,000 runs. Four, five, and eight were the most likely number of clusters for the slum, intermediate, and city‐wide scales, respectively. At the slum scale, most individuals in valleys 1 and 4 were clustered together, while Valley 2 individuals were clearly separated into a distinct group (Figure 2c). At the intermediate scale, the patterns differed slightly; Valley 4 was subdivided into two groups that were clustered away from valleys 1 and 2 (Figure 3c). Some satellite sites were grouped with other satellites, while others were included with the Pau da Lima valley clusters. At the broadest city‐wide scale, the samples from the three Pau da Lima valleys were genetically isolated to their own valley (Figure 4c). The two sites in the southwestern part of the city (FSJ, LP) cluster together around the port, while other satellite sites represent their own group or cluster with either Valley 1 or Valley 4 (Figure 4c).

### 3.4 Moran's eigenvector mapping to account for spatial nonindependence

Moran's eigenvector analysis indicated that the proportion of all genetic variation attributed to spatial patterns differed across scales. The smallest scale (within slum) had the most genetic variation that was explained by spatial patterns (adjusted r^2 ^= .124), representing a surprisingly high degree of spatial genetic structure at such a small scale, given that the area is only 0.37 km^2^. Genetic variation at the intermediate (r^2 ^= .051) and city‐wide scales (r^2 ^= .06) was less strongly shaped by spatial orientation. At all scales, the first MEM variable, representing the principal coordinate eigenvector, explained more than 30% of the spatial genetic variation (proportional variances: 0.30 for the slum scale, 0.33 for the intermediate scale, and 0.41 for the city‐wide scale). Visualizing this first MEM variable score within Pau da Lima indicates that valleys 2 and 4 are strongly divergent despite their adjacent locations (Figure 2d). Valley 1 is genetically intermediate, with MEM scores closer to Valley 4 than Valley 2 (Figure 2d). MEM scores at the intermediate and city‐wide scales highlight the genetic distinction between Pau da Lima rats and all other sites across Salvador (Figures 3, and 4d).

### 3.5 Migration rates across Salvador

As expected, most of the recent gene flow (i.e., over the last several generations) occurred within sites, rather than among sampled areas. An average of 79% of “migration” occurred internally within each valley, satellite site, or sampling area. Valley 2 showed the greatest genetic insulation with 90% of individuals identified as originating from within Valley 2. In Valley 4, 84% of individuals were estimated to have originated within this valley. Consistent with the other spatial genetic analyses, Valley 1 showed the highest proportion of migrants from outside this valley (71% from within Valley 1). The source of migration estimated to have come into Pau da Lima, and other sampling areas, was distributed fairly evenly across the other sampling areas (i.e., no single area contributed disproportionately high migration rates into any other population). A Mantel matrix correlation test found no significant relationship between migration rate and geographic distance among sampled areas (r = −.116, *p *=* *.96), or pairwise migration rate and genetic distance (r = −.13, *p *=* *.98). There was a significant association between geographic and genetic distance among sampling groups across the city, indicating isolation by distance in this system (r = .451, *p *=* *.008).

## 4 Discussion

Norway rats within the Pau da Lima slum showed strong patterns of genetic divergence within a very small area, despite modest divergence across the entire city of Salvador, Brazil. Importantly, this pattern of fine‐scale divergence was consistent using four very different analytical approaches and spatial scales. Within Pau da Lima, a sharp break in genetic similarity distinguished valleys 2 and 4, two geographically distinct micro‐districts within this densely inhabited slum settlement. Our migration data also indicate that rat movements occur mostly within their home valley or sampling neighborhoods and that there may not be clear “source” populations contributing to the bulk of migrants. Together, we use these spatial genetic data to identify units of eradication that are practical for future intervention campaigns.

### 4.1 Genetic patterns at three spatial scales

Analyses at the smallest scale incorporated all 493 rats sampled within the Pau da Lima community (Figure 1). The most striking pattern at this spatial scale is the large degree of genetic divergence among rats located within half a kilometer of each other. The sharpest genetic breaks occur among rats in valleys 2 and 4, separated by <50 m (Figure 2a–d). This is remarkable considering that the average dispersal distance for urban *R. norvegicus* is 150 m (Glass et al., 1989; Traweger, Travnitzky, Moser, Walzer, & Bernatzky, 2006; Richardson unpublished data) and more than 1 km for *R. norvegicus* in rural areas (Macdonald & Fenn, 1995; Taylor & Quy, 1978). Given these dispersal abilities, our data indicate that there is a strong barrier to movement and gene flow between valleys 2 and 4. These two valleys are separated by a modest saddle, representing 50 m of elevation gain, as well as a high‐traffic road connecting two neighborhoods on either side of Pau da Lima. Rat movements are likely impeded by the combination of this road and topographic relief.

The intermediate‐scale analyses included 615 rats from Pau da Lima and the eight satellite sites sampled within 1 km of Pau da Lima. The strongest signal of genetic divergence at this scale was again between valleys 2 and 4 within Pau da Lima (Figure 3a–c). The association between Valley 1 and the rest of Pau da Lima was less consistent, with Valley 1 more similar to Valley 2 for the DAPC and Bayesian model approaches (Figure 3b,c), but showing admixture more closely associated with Valley 4 in the sPCA (Figure 3a). Interestingly, the level of admixture for Valley 1 switched from being more associated with Valley 2 at the slum scale or Valley 4 at this intermediate scale. This was true for both the sPCA (Figures 2a, and 3a) and spatial Bayesian model (Figures 2c, and 3c). These results indicate that valleys 2 and 4 are strongly diverged genetically, while Valley 1 is admixed with affinities to both valleys 2 and 4. As noted above, valleys 2 and 4 are separated by an altitudinal saddle and a busy road, but the immediately adjacent valleys 1 and 2 are separated by only a low‐lying area, inundated with water during the rainy winter months, with few human settlements (Figure 1b). A model‐based approach (e.g., approximate Bayesian computation) may be able to distinguish between true admixture and other mechanisms, such as drift, that could create the inconsistent pattern observed for Valley 1. The intermediate‐scale MEM analysis showed very little divergence among all Pau da Lima samples, but separation between Pau da Lima and the nearby satellite sites (Figure 3d). MEM analyses are expected to be particularly useful at small scales where higher gene flow occurs, such as within genetically distinct groups (Galpern et al., 2014). The lower sensitivity of MEM between genetically divergent groups may explain why MEM is consistent with the other analyses at the Pau da Lima scale (Figure 2), but does not distinguish the separate valleys at the two larger scales where the genetic divergence is much greater between Pau da Lima and the satellite sites (Figures 3d, and 4d, Tables S1 and S2).

When analyzing all 706 rat samples from the city‐wide scale, which included rats from 17 sites across Salvador, the sites situated further from the intermediate‐scale sampling area were more similar to each other than to the sites at the smaller spatial scales (Figure 4a,b,d). Consistent with the results for the other two spatial scales analyzed, Valley 2 is genetically distinct from valleys 1 and 4 within Pau da Lima in the sPCA (Figure 4a), with the DAPC analysis clearly segregating Valley 4 from all other sites (Figure 4b). The approach using a spatially explicit Bayesian model did identify distinct genetic groups among the city‐wide sampling sites (Figure 4c), where sites located outside the Pau da Lima slum and intermediate scales were each assigned to distinct genetic groups, with the exception of two sites in the southwestern area of the city (FSJ and LP). These sites are the closest pair among the outlying Salvador sites, located <3 km apart. Additionally, they are both in close proximity (within 3 km) to Salvador's main port—the most likely point of entry for additional genotypes of newly introduced rats. This Bayesian model also identified the micro‐scale differentiation within the Pau da Lima slum (Figure 4c).

### 4.2 Using spatial genetic patterns to guide public health interventions

Our study provides important insights into the movement and spatial genetic patterns of Norway rats in Salvador, Brazil. These insights are timely, as the ministry of health is planning intervention campaigns to reduce the prevalence of leptospirosis in Pau da Lima and other sections of Salvador by wide‐scale application of rodenticides. Similar genetic data have been used to outline eradication units in studies concerned with controlling island pest species (Adams, van Heezik, Dickinson, & Robertson, 2014; Piertney et al., 2016; Ragionieri et al., 2013; Robertson & Gemmell, 2004). More broadly, the methods used in the current study provide important advances in both sampling and analysis that can benefit future studies of urban epidemiology and vector control.

Strong spatial genetic patterns can be used to infer levels of movement and connectivity across a study area, and delineate unique genetic groups that can be treated as focal units during intervention campaigns. Epidemiologists and public health officials often define “eradication units” as interconnected groups of vector organisms that are targeted during control efforts. Defining eradication units also makes evaluating the effectiveness of an intervention campaign possible in a tractable way (Robertson & Gemmell, 2004). For example, rigorous estimates of rat population sizes, densities, and removal rates can be estimated for each of the defined units to monitor the efficacy of control campaigns (Hacker et al., 2016). Genetic data can provide another important facet of eradication success when samples can be compared before and after intervention campaigns to evaluate the degree of genetic variation that is lost or reduced via postintervention genetic bottlenecks (Abdelkrim, Pascal, Calmet, & Samadi, 2005; Russell et al., 2010; Veale et al., 2013).

In the current study, we found strong genetic divergence between valleys in Pau da Lima. This divergence was strong and consistent between rats in valleys 2 and 4, located <50 m apart. Valley 1 was also divergent from the other two valleys, but this pattern differed across analyses and scales suggesting higher admixture in Valley 1. These spatial genetic patterns support defining eradication units based on these valley divisions within Pau da Lima. Specifically, the genetic data strongly support valleys 2 and 4 being delineated as separate units. The fine‐scale DAPC and MEM analyses also support the segregation of Valley 1 from both other valleys as another eradication unit (Figure 2b,d). Moreover, we identified that high‐traffic roads and modest topography likely play a role in impeding rat movement and that both features can be used to define eradication units in the context of Salvador. Additionally, the observed migration rates indicate that most rat movements (71–90%) are contained within valleys, providing further support for creating eradication units based on valleys. It is important to note that using topography alone would not be sufficient, as valleys 1 and 2 are connected via a low‐elevation area. The spatial genetic data provide clear and consistent results across analyses to use the valley boundaries to define eradication units and design future intervention campaigns. However, an explicit landscape genetics analysis is needed to identify the exact features shaping the spatial genetic patterns observed.

We focused genetic sampling on the areas within and around Pau da Lima because this slum has been the focus of long‐term epidemiological research (Felzemburgh et al., 2014; Hagan et al., 2016; Maciel et al., 2008). Pau da Lima has high human and rat densities, poor municipal infrastructure, and high incidence of leptospirosis cases corresponding to elevated levels of *Leptospira* infection exceeding 80% among Norway rats in this area (Costa et al., 2014; Felzemburgh et al., 2014). *Rattus norvegicus* is the primary host reservoir, and control campaigns are conducted regularly to target rats for removal. However, rat populations rebound in size within 6–12 months, as indicated by surveys of rat activity and abundance (Hacker et al., 2016). Therefore, identifying the contributions of remnant eradication survivors vs. immigrant re‐colonizers to this rapid recovery is a central goal in the public health strategies designed to reduce leptospirosis in urban centers. Distinguishing between these sources is critical to determining the efficacy of the control campaign, and if future improvement is more likely to come from increased local interventions or continuous targeting of dispersal corridors (Russell et al., 2010). Our data indicate that spatial genetics and migration assignment techniques can be used to differentiate the source of new animals postintervention. While the current study does not focus on pre‐ and postintervention sampling, our migration data suggest that most individuals originated from within their specific sampling area. Further, there does not appear to be a clear source population contributing any sizeable proportion of identified migrant rats. However, future studies would need to explicitly sample for pre‐ and postintervention genetic patterns to suitably address this within Pau da Lima.

There are practical and immediate applications of these data in designing future intervention campaigns in Salvador. These data provide a better understanding of where rats are moving in a dense urban landscape and which areas constitute genetically, and presumably demographically, isolated units. In the case of Pau da Lima and the surrounding area, we recommend units corresponding to valley designation and satellite sampling neighborhoods, based on our data. The ministry of health in Salvador is using these units as the focal areas to delineate the boundaries of future intervention campaigns. This means that instead of spreading resources (i.e., traps, manpower) across all of Pau da Lima, particular valleys can be targeted individually. Moreover, continual trapping efforts can be used to limit rats migrating between valleys 1 and 2, even outside of intervention campaigns. Our data suggest that these permanent trapping efforts are not needed between valleys 2 and 4. Importantly, verifying the success of this modified intervention strategy will require evaluating rat numbers and genetic signatures before and after control campaigns, as described in the previous paragraph.

### 4.3 Advancing spatial genetic studies in urban contexts

Our study demonstrates the power and utility of combining dense sampling with spatially explicit analyses to characterize the genetic structure and likely movement patterns of wildlife in complex urban landscapes. These patterns are difficult to study, but Norway rats are an excellent species to investigate urban ecology and movement. *Rattus norvegicus* is a globally distributed pest species invasive to most cities because it is so adept at exploiting urban habits and resources (Long, 2003). This species has been the focus of many ecological and epidemiological studies in cities around the world (Capizzi et al., 2014); however, few studies have looked at the genetic patterns of movement and gene flow within an urban context. Gardner‐Santana et al. (2009) evaluated genetic structure at 10 microsatellite loci for 277 rats from 11 sites within ~84 km^2^ of Baltimore, Maryland, USA. Three genetic groups were distinguished based on Bayesian clustering and high assignment probabilities, which suggested low levels of dispersal among the 11 separate sampling locations, despite examples of rare long‐distance dispersal (Gardner‐Santana et al., 2009). A previous study of 146 Norway rats captured within and around Pau da Lima found genetic divergence among three identified genetic clusters from nine sites (Kajdacsi et al., 2013). The current study extends the findings of Kajdacsi et al. (2013) by including many more rats (*n* = 706) captured over a much wider area across Salvador. These previous studies from Baltimore and Salvador support our findings that genetic heterogeneity exists within urban landscapes, even at very small scales. However, the two aforementioned studies were limited by the low density of samples across complex urban landscapes.

Our use of individual‐based analyses is an important advance in spatial population genetics. Individual‐based approaches treat individual samples as the unit of analysis, negating the need to group samples *a priori* for analysis, which is often arbitrary with respect to biology (Waples & Gaggiotti, 2006). With sufficient sampling density, this analytical framework can also be more powerful in detecting genetic patterns at small spatial scales (Luximon et al., 2014; Prunier et al., 2013). This increased power to detect fine‐scale genetic patterns is particularly useful when investigating urban landscapes, where habitat complexity such as density of humans and the presence of physical barriers (e.g., roads and topographical features) can be much higher than exurban areas (Pickett et al., 2008). Population densities of urban species can also be inflated relative to natural densities, facilitating the sampling density needed for individual‐based analyses. To this effect, we were able to collect and analyze a large numbers of samples (*n* = 493) within a relatively small area within the Pau da Lima neighborhood (0.37 km^2^), which vastly exceeds previous efforts. Such high‐resolution spatial genetic data provide newly available and powerful information for the public health initiatives to control Norway rats and advance urban population genetics more broadly.

Very few studies have explicitly investigated how scale can influence observed spatial genetic patterns. Those that have evaluated scale found similar patterns to what we observed in Salvador rats, including different patterns of genetic divergence in simulated (Cushman & Landguth, 2010) and empirical data using amphibians (Angelone, Kienast, & Holderegger, 2011) and insects (Keller, Holderegger, & van Strien, 2013). However, none of these previous studies investigated urban landscapes, where high habitat heterogeneity exists at much finer scales (Hacker et al., 2013; Pickett et al., 2008). In our study, most analyses retained patterns of spatial genetic divergence across valleys at different scales. The specific genetic groupings of rats in Salvador varied among the different scales of analysis based on the analytical methods. For example, sPCA and the Bayesian analyses have Valley 1 associated with Valley 2 at one scale and with Valley 4 at another scale (Figure 2a,c and Figure 3a,c). However, one pattern that was consistent across all scales and analyses was the clear divergence between valleys 2 and 4. These multiscale analyses illustrate the importance of either defining the scale of investigation based on detailed knowledge or specific hypotheses about mechanisms of genetic divergence over space (Richardson et al., 2016) or explicitly evaluating patterns at multiple spatial scales to identify how patterns differ. Either approach requires a sampling design with sufficient power to detect spatial genetic patterns (Oyler‐McCance, Fedy, & Landguth, 2013).

It should be noted that genetics is a powerful proxy for dispersal and movement of individual across a landscape; however, genetic data only represent migrants and movements that result in successful breeding. Therefore, individual movements that do not lead to breeding and genetic exchange are not detected by genetic data, biasing connectivity estimates downward (Lowe & Allendorf, 2010). The dense sampling employed in the current study is one way to minimize the risk of not detecting nonbreeding dispersers. We also estimated migration rates, which are designed to detect the movement of first‐generation migrants regardless of their breeding status. So a scenario where a large proportion of rats are moving across Salvador or Pau da Lima but not breeding (e.g., if high densities of rats with strong priority effects are preventing migrants from penetrating new areas; Fraser et al., 2015) is possible, but very unlikely given our study design and sharp patterns of genetic divergence. In fact, the sharp genetic boundaries we detected are all the more striking if previous intervention campaigns disrupted priority effects and competitive exclusion of migrants by resident rats.

### 4.4 Potential role of evolution in urban rat populations

The observed patterns of strong genetic divergence at very fine spatial scales may arise not only from restricted movement and gene flow, but also from evolutionary divergence at microgeographic scales. This could occur if environmental conditions differ substantially within a small area such as Pau da Lima's valleys. While speculative for rats in Salvador, evidence from other studies indicates that complex urban habitats often impose strong and diverse natural selection on urban‐dwelling species (Donihue & Lambert, 2015). Dissimilar habitats can promote divergent natural selection on rats occupying different parts of the environmental space, creating the potential for adaptive divergence within a small area (Richardson et al., 2014). For example, strong divergence in urban rodents has been linked to environmental differences in New York City, where genetic differences linked to metabolic function and dietary specialization have arisen within decades, rather than centuries (Harris & Munshi‐South, 2016; Harris, Munshi‐South, Obergfell, & O'Neill, 2013).

We do not currently know enough about the evolutionary selective environment for urban rats, and how this differs across Salvador, to ascribe the observed genetic patterns to evolutionary divergence. However, the sharp break in genetic similarity between Valley 2 and Valley 4 indicates the potential for such adaptive divergence to either arise or explain the observed genetic break. Outbreeding avoidance may also contribute to this divergence, where rats are more likely to breed with more closely related individuals coming from within the local population, rather than immigrants from other areas of Pau da Lima or Salvador (Costa et al., 2016). There are an increasing number of examples of evolutionary divergence at microgeographic scales, including in response to complex urban environments and their natural selection regimes (Richardson et al., 2014; Saccheri, Rousset, Watts, Brakefield, & Cook, 2008; Selander & Kaufman, 1975).

## 5 Conclusions

High‐resolution spatial genetic data can provide important information for epidemiological studies on the areas of movement, gene flow, and potential routes of recolonization after interventions to control vector species. Here, we used such data on over 700 rats in Salvador Brazil to (A) define eradication units based on genetic divergence among valleys in the Pau da Lima slum community, (B) evaluate migration rates to determine that no clear source area is responsible for recolonization after intervention campaigns that reduce rat populations, and (C) advance the methods associated with urban ecology using spatially explicit and individual‐based approaches to evaluate genetic patterns at multiple spatial scales. The data from this study are particularly useful when combined with other epidemiological interventions, including reductions in the number of environmental reservoirs supporting the parasite, and implementing changes in human behavior that can reduce the prevalence of disease. These approaches are currently being employed in urban centers in Brazil and other developing countries to reduce the risk of leptospirosis, which has emerged due to the expansion of slum settlements and rat populations that populate such communities. The approaches used in this study also provide a framework for future work on urban ecology and landscape genetics, as well as the potential for microgeographic evolution in urban habitats that vary dramatically within very small spatial scales, in terms of both landscape features and genetic divergence.

## Data Archiving Statement

Data for this study are included as Genepop‐formatted genotypes in a supplemental file in the online version of this article.
