# Supplementary material for: Using fine‐scale spatial genetics of Norway rats to improve control efforts and reduce leptospirosis risk in urban slum environments
Source: Evol Appl. 2017 Feb 23;10(4):323–37. doi: 10.1111/eva.12449 (PMC5367079; doi:10.1111/eva.12449)

**Supporting Information**

**Figure S1.** A map of the 28 geographic groups used to make estimating pairwise migration computationally feasible. We grouped the 706 rats based on topography and distance from other sampling areas.


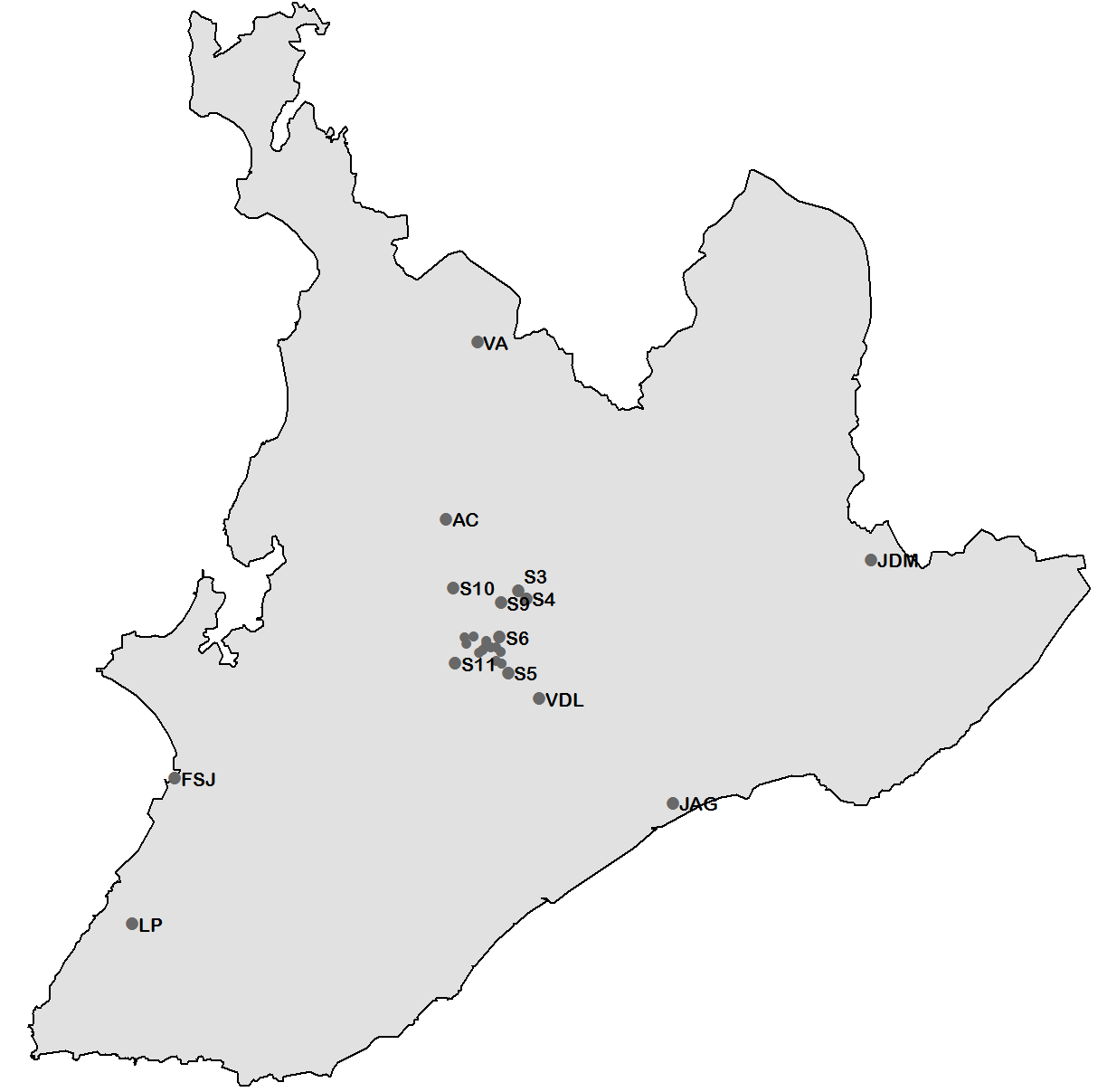

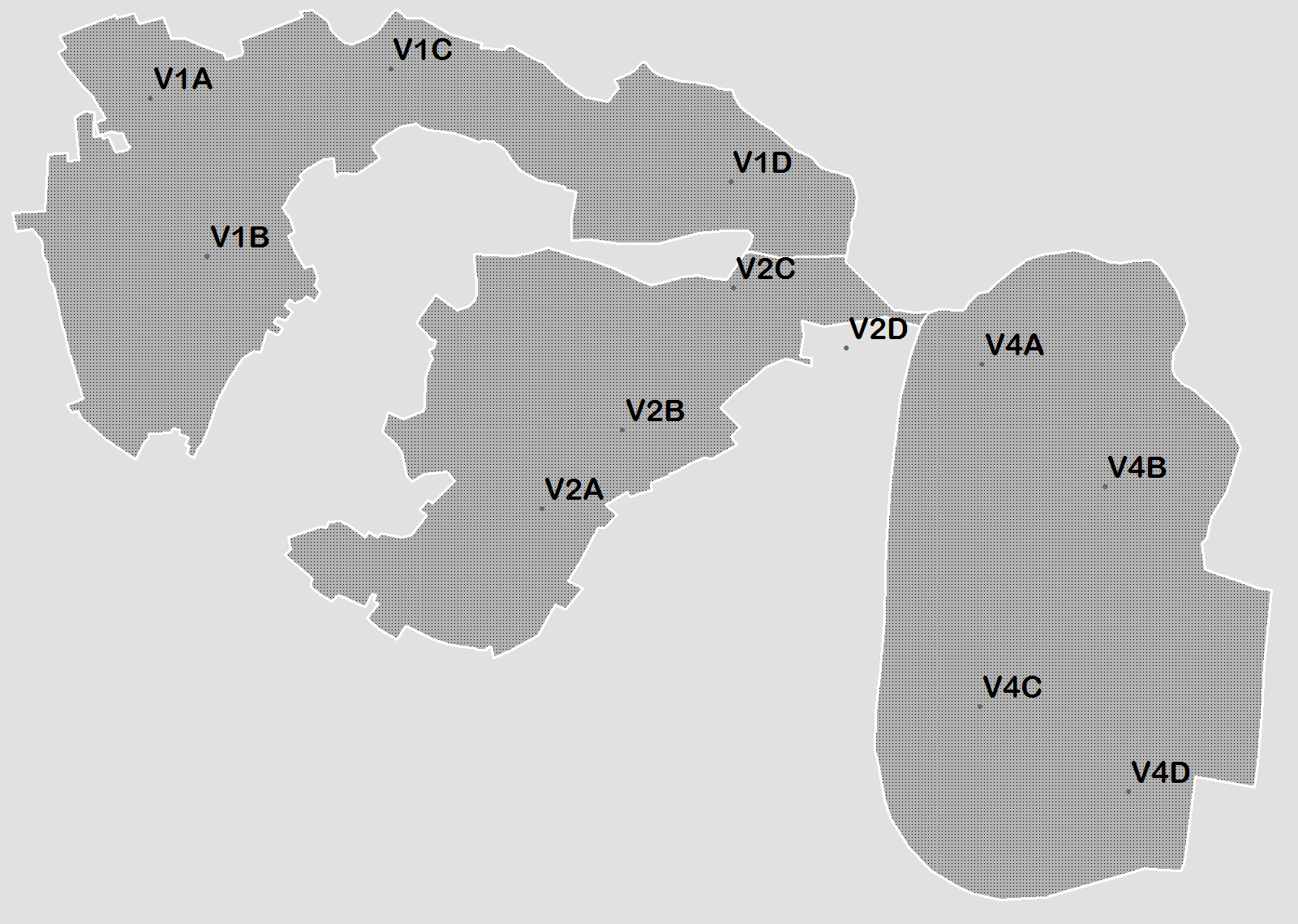


**Summary statistics and marker vetting:**

*** Note that we provide the following population-level estimates of Fst and HWE only for comparison with commonly used population-level analyses; however rats in our urban context are distributed more uniformly than discrete “populations”. We use individual-based analyses in the current study to avoid bias arising from deviations from the island models that population-level analyses are based on.*

There was very little missing data in our 706 genotypes, representing 6.1% off allele calls.

Global Fst values across all sites were 0.093 at the city-wide scale, 0.064 at the intermediate scale, and 0.049 at the Pau da Lima slum scale. Pairwise Fst values ranged from near zero to 0.44 (see Tables S1 and S2 below).

There were 2,160 combinations of loci across the 18 sampling sites (120 unique locus pairs x 18 sites) that were tested for linkage disequilibrium. There was only evidence of linkage disequilibrium (LD) in 14.9% of these combinations, but there was no consistent LD across sites. No locus pair was in LD in more than 5 of the 18 sites, indicating that the loci analyzed were not physically linked with each other.

Hardy-Weinberg equilibrium (HWE) was assessed for each population for each locus. Four of the 18 sampling sites showed evidence of departing from HWE, but not systematically across loci. The 4 sites only had between 1 and 5 loci (median of 2) out of HWE. For this reason, and the fact that our species’ distribution is not discrete and our analyses do not assume HWE, we retained all 16 loci for the final analyses.

**Table S1.** Pairwise Fst values between each sampling location at the Salvador-wide scale. Values significantly greater than zero are denoted with bold text. ** Note that we provide these Fst values only comparison with population-level analyses; however rats in our urban context are distributed more uniformly than discrete “populations”. We use individual-based analyses in the current study to avoid bias arising from deviations from the island models that population-level analyses are based on.

|  | V1 | V2 | V4 | S5 | S6 | S9 | S10 | S11 | VDL | S3 | S4 | FSJ10 | LP | VA | AC | JAG | JDM | FSJ14 |
| --- | --- | --- | --- | --- | --- | --- | --- | --- | --- | --- | --- | --- | --- | --- | --- | --- | --- | --- |
| V1 | 0.000 | **0.020** | **0.041** | **0.041** | **0.037** | **0.048** | **0.142** | **0.020** | **0.119** | **0.060** | 0.075 | 0.144 | **0.125** | **0.138** | **0.171** | **0.241** | **0.161** | **0.116** |
| V2 | **0.020** | 0.000 | **0.044** | **0.046** | 0.029 | **0.059** | **0.139** | **0.017** | **0.121** | **0.049** | 0.053 | 0.130 | **0.109** | **0.128** | **0.161** | **0.231** | **0.161** | **0.111** |
| V4 | **0.041** | **0.044** | 0.000 | **0.062** | 0.032 | **0.077** | **0.160** | **0.053** | **0.154** | **0.093** | 0.109 | 0.097 | **0.115** | **0.116** | **0.143** | **0.198** | **0.131** | **0.091** |
| S5 | **0.041** | **0.046** | **0.062** | 0.000 | 0.052 | **0.085** | **0.184** | 0.025 | **0.135** | **0.085** | 0.092 | 0.151 | **0.137** | **0.140** | **0.185** | **0.266** | **0.155** | **0.133** |
| S6 | **0.037** | 0.029 | 0.032 | 0.052 | 0.000 | 0.084 | **0.185** | 0.036 | **0.170** | 0.078 | 0.052 | 0.113 | 0.114 | **0.130** | 0.114 | **0.269** | 0.112 | **0.109** |
| S9 | **0.048** | **0.059** | **0.077** | **0.085** | 0.084 | 0.000 | **0.227** | **0.078** | **0.226** | **0.101** | 0.113 | 0.211 | **0.135** | **0.145** | **0.217** | **0.334** | 0.192 | **0.147** |
| S10 | **0.142** | **0.139** | **0.160** | **0.184** | **0.185** | **0.227** | 0.000 | **0.144** | **0.280** | **0.184** | 0.280 | 0.331 | **0.270** | **0.294** | **0.318** | **0.442** | 0.280 | **0.187** |
| S11 | **0.020** | **0.017** | **0.053** | 0.025 | 0.036 | **0.078** | **0.144** | 0.000 | **0.123** | **0.051** | 0.066 | 0.134 | **0.096** | **0.145** | **0.159** | **0.283** | **0.147** | **0.110** |
| VDL | **0.119** | **0.121** | **0.154** | **0.135** | **0.170** | **0.226** | **0.280** | **0.123** | 0.000 | **0.206** | 0.220 | 0.299 | **0.256** | **0.267** | 0.260 | 0.392 | 0.265 | **0.212** |
| S3 | **0.060** | **0.049** | **0.093** | **0.085** | 0.078 | **0.101** | **0.184** | **0.051** | **0.206** | 0.000 | 0.082 | 0.212 | **0.169** | **0.201** | **0.226** | **0.330** | 0.226 | **0.157** |
| S4 | 0.075 | 0.053 | 0.109 | 0.092 | 0.052 | 0.113 | 0.280 | 0.066 | 0.220 | 0.082 | 0.000 | 0.234 | 0.194 | 0.214 | 0.188 | 0.447 | 0.168 | 0.172 |
| FSJ10 | 0.144 | 0.130 | 0.097 | 0.151 | 0.113 | 0.211 | 0.331 | 0.134 | 0.299 | 0.212 | 0.234 | 0.000 | 0.100 | 0.179 | 0.120 | 0.388 | 0.123 | 0.086 |
| LP | **0.125** | **0.109** | **0.115** | **0.137** | 0.114 | **0.135** | **0.270** | **0.096** | **0.256** | **0.169** | 0.194 | 0.100 | 0.000 | **0.141** | 0.151 | **0.319** | 0.126 | **0.110** |
| VA | **0.138** | **0.128** | **0.116** | **0.140** | **0.130** | **0.145** | **0.294** | **0.145** | **0.267** | **0.201** | 0.214 | 0.179 | **0.141** | 0.000 | **0.144** | **0.281** | **0.129** | **0.144** |
| AC | **0.171** | **0.161** | **0.143** | **0.185** | 0.114 | **0.217** | **0.318** | **0.159** | 0.260 | **0.226** | 0.188 | 0.120 | 0.151 | **0.144** | 0.000 | **0.341** | 0.091 | **0.184** |
| JAG | **0.241** | **0.231** | **0.198** | **0.266** | **0.269** | **0.334** | **0.442** | **0.283** | 0.392 | **0.330** | 0.447 | 0.388 | **0.319** | **0.281** | **0.341** | 0.000 | 0.300 | **0.293** |
| JDM | **0.161** | **0.161** | **0.131** | **0.155** | 0.112 | 0.192 | 0.280 | **0.147** | 0.265 | 0.226 | 0.168 | 0.123 | 0.126 | **0.129** | 0.091 | 0.300 | 0.000 | **0.146** |
| FSJ14 | **0.116** | **0.111** | **0.091** | **0.133** | **0.109** | **0.147** | 0.187 | **0.110** | **0.212** | **0.157** | 0.172 | 0.086 | **0.110** | **0.144** | **0.184** | **0.293** | **0.146** | 0.000 |

**Table S2.** Pairwise Fst values between each sampling location at the intermediate scale. Values significantly greater than zero are denoted with bold text. ** Note that we provide these Fst values only comparison with population-level analyses; however rats in our urban context distributed more uniformly than discrete “populations”. We use individual-based analyses in the current study to avoid bias arising from deviations from the island models that population-level analyses are based on.


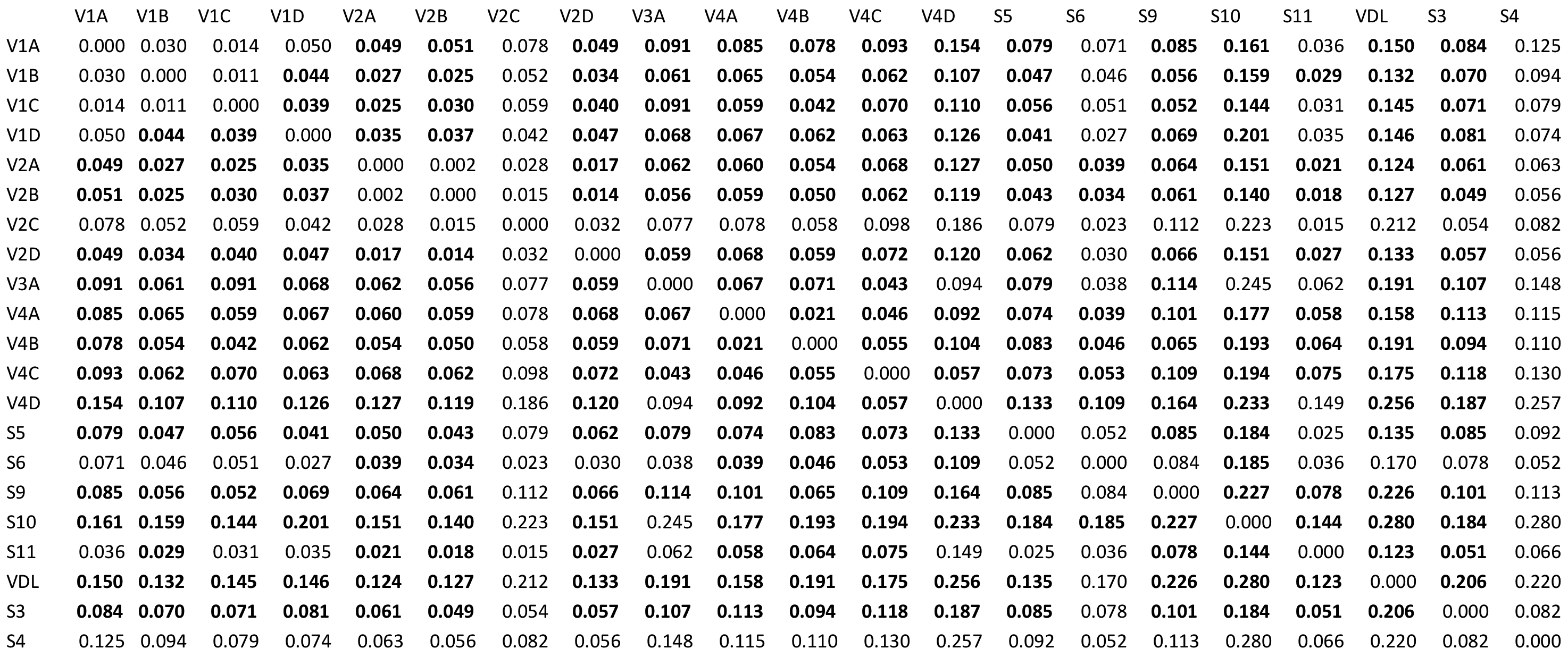

Supplement: Supplementary file 1 [file EVA-10-323-s001.docx]
